# The Effect of Bone Marrow Mesenchymal Stem Cells on Vitamin D3 Induced Monocytic Differentiation of U937 Cells

**DOI:** 10.15171/apb.2016.05

**Published:** 2016-03-17

**Authors:** Zahra Molaeipour, Karim Shamsasanjan, Ali Akbari Movassaghpour, Parvin Akbarzadehlaleh, Fatemeh Sabaghi, Mahshid Saleh

**Affiliations:** ^1^ Hematology Oncology Research Center, Tabriz University of Medical Sciences, Tabriz, Iran.; ^2^ Pharmaceutical Biotechnology Department, Tabriz University of Medical Sciences, Tabriz, Iran.; ^3^ Blood Transfusion Research Center, High Institute for Research and Education in Transfusion Medicine, Tehran, Iran.

**Keywords:** Mesenchymal stem cells, U937 cells, Differentiation, Vitamin D3

## Abstract

***Purpose:*** Mesenchymal stem cells (MSCs) are key components of the hematopoietic stem cells (HSCs) niche. They control the process of hematopoiesis by secreting regulatory cytokines, growth factors and expression of important cell adhesion molecules for cell-tocell interactions. In this research, we have investigated the effect of bone marrow derived MSCs on monocytic differentiation of U937 cells line.

***Methods:*** U937 cells were cultured in both direct co-culture with MSCs and MSCs conditioned medium (C.M) driven. This study used 1,25-dihydroxyvitamin D3(VitD3) as inductor of monocytic differentiation and U937 cells treated with VitD3 morphology was examined by Wright Giemsa staining. CD14 monocytic differentiation marker was measured by flow cytometry and monocytic gene expression was assessed by real time polymerase chain reaction (RT PCR).

***Results:*** The results of flow cytometric analysis showed that CD14 expression of U937 increased. The higher effect of MSCs co-culture on CD14 expression in U937 cells was observed, compared to the conditioned medium. Among ten monocytic related genes which were screened that was observed increase in 5 genes in which CXCR4 and CSF2RA showed significant increase.

***Conclusion:*** The results obtained show that MSCs have supportive effect on the monocytic differentiation of U937 cells. However, a distinct mechanism of that remains unclear.

## Introduction

The bone marrow contains hematopoietic cells and non-hematopoetic cells such as mesenchymal stem cells (MSCs).^1^ In addition to bone marrow, MSCs‏ are found in adipose tissue, amniotic fluid, synovial tissue, skeletal‏ muscle, heart, placenta, cord blood and circulating blood.^2^ The International Society of Cellular Therapy (ISCT) proposed the minimal criteria to define human MSCs. These criteria include adherence to plastic in standard culture, expression of CD105, CD73, and CD90, lack of the expression of hematopoietic markers and differentiation potential into osteocytes, adipocytes, and chondrocyte.^3^ MSCs can differentiate into several types of cells and produce important growth factors and cytokines.^4^ MSCs are main components of the hematopoietic stem cell (HSC) niche, where the stem cells are housed.^5^ MSCs are involved in the regulation of hematopoietic precursor cells proliferation and differentiation.^6^ When HSCs are in close proximity to the MSCs, they become attached via adhesion molecules such as N-cadherin and *β* -integrins. The Wnt pathway from HSC upregulates the Notch ligands (Jagged and Delta-like) in MSC and activates the Notch signaling pathway in HSC.^7^ Bone marrow-derived MSCs secrete several cytokines and growth factors such as the stem cell factor (SCF), CXCL12 (SDF-1), granulocyte-macrophage colony-stimulating factor (GM-CSF), macrophage colony-stimulating factor (M-CSF), Flt-3 ligand (FL), interleukin (IL)-6, IL-11, thrombopoietin (TPO), tumor necrosis factor- (TNF-) *α*, and transforming growth factor- (TGF-) *β*1.^8^ GM-CSF stimulates proliferation and differentiation of macrophage progenitors at the lowest doses, followed by granulocyte, eosinophil, erythroid, megakaryocyte and multipotent progenitors.^9^ M-CSF (CSF-1) controls the proliferation, differentiation, and survival of monocytes, macrophages and bone marrow progenitor cells.^10^ MSCs have many therapeutically advantageous features such as easy acquisition, fast *ex vivo* expansion and no immunogenic effects and therefore they can be utilized in clinical regenerative medicine.^11^ Differentiation therapy is an approach to treating malignant diseases. This approach employs agents that modify cell differentiation.^12^ 1,25-dihydroxyvitamin D3(VitD3) could play a role in the treatment of acute myeloid leukemia (AML) and some hematologic disorders.^13^ VitD3 has been recognized to induce differentiation of myeloid leukemic cells including the myelomonocytic U937 cell line, through growth inhibition.^14^

The effects of various cells in the bone marrow niche are unclear on hematopoietic stem cells differentiation, and MSCs as precursors of the cellular components, are important cells of the bone marrow niche. In this study we have investigated the effect of bone marrow mesenchymal stem cell on differentiation of U937 cells, a myelomonocytic leukemia cell line to monocytic lineage.

## Materials and Methods

### Cell culture

The U937 cells, a human myelomonocytic leukemia cell line (kind gift from Dr. Abroun, Tarbiat Modares University, Tehran, Iran), cultured in RPMI-1640 medium (Sigma-Aldrich, USA) with 100 U/ml penicillin, 100μg/ml streptomycin (Gibco, UK) and 10% fetal bovine serum (FBS, Gibco, UK) was incubated in humidified incubator with 5% CO_2_ at 37°C. Cells were allowed to pass through every 2 to 3 days to maintain a log phase growth. The cell viability was evaluated by trypan blue staining. Bone marrow-derived MSCs, were purchased from StemCell Technology, Tehran, Iran, that confirmed the antigens CD73, CD90, CD105 positive, CD11b, CD14, CD19, CD34, CD45, CD79a, HLA-DR negative, by flow cytometry. MSCs were removed by 0.04% Trypsin/0.03% EDTA and cultured in Dulbecco's Modified Eagle Medium (DMEM)-LG (Gibco, UK), containing 10% fetal bovine serum and incubated at 37°C and 5% CO_2_. The cells went through when they reached 80% confluence .We used the third passage cells all through the experiments.The study groups arranged as follows: Group I : U937 cells without treating as control group. Group II : U937 cells treated with Vit D3 as Positive Control, Group III : Co-culture of U937 with MSC and VitD3, Group IV : U937 cells cultured in conditioned medium with VitD3.

### Cell treatment

VitD3 (1*α*,25-dihydroxyvitamin D3; 1,25D) has been recognized to induce differentiation of U937 cell line.^14^ U937 cells (1×10^6^/ml) containing 5×10^-8^ M concentration of VitD3 (Sigma-Aldrich, USA) were used as positive control.VitD3 concentration was determined on the basis of our previous works. Medium was changed on the third day and VitD3 concentration was maintained.

### Morphological study of differentiated monocytic cells

To study changes in differentiated U937 cells treated with VitD3, the Wright Giemsa stain was performed and studied by light microscope.

### U937 cells co-culture with MSCs

In this study direct cell-cell contact co-culture method have been used. MSCs (1×10^4^/cm^2^)^2^ were seeded into a culture flask and when 60% confluence was achieved, U937 cells (1×10^6^/ml) along with 5×10^-8^ M concentration of VitD3 were added and incubated at 37°C in 5% CO_2_. RPMI-1640 medium has been used for U937 cells co-culture with MSCs.

### Culture of U937 cells with conditioned medium from MSCs

The MSCs were cultured RPMI-1640 medium for 24 hours in 5% CO_2_ at 37°C, then the culture medium was centrifuged and stored at -80°C to be used as conditioned medium. U937 cells were cultured in the conditioned medium with 5×10^-8^ M concentration of VitD3 at 37°C, with 5% CO_2_.

### Flow cytometric assessment of monocytic marker of differentiation

U937 cells (1*×*10^6^/ml) related to four groups, after 24 hours were harvested and centrifuged at 3500g for 5 minutes at room temperature then washed with phosphate buffer saline (PBS) and incubated with anti-CD14 PE (Becton Dickinson, USA) for 30 minutes at 4°C. After washing twice with PBS, the cells were suspended in PBS and analyzed for evaluation of the monocytic marker CD14 expression by a flow cytometer (FACS Calibor, USA).

### Real-Time Polymerase Chain Reaction (RT-PCR)

The expression of monocytic genes was investigated by Real-Time PCR after 24 hours incubation. Total RNA was extracted using QIAzol lysis reagent (QIAGEN, USA) according to the manufacturer’s instructions. RNA quality was evaluated by the spectrophotometric absorbance ratio at 260/280 nm (Picodrop, Uk). cDNA was prepared according to BioRT cDNA first strand synthesis Kit protocol (Bioer, Japan).

CD14, CXCL8, TNFRSF11A, CSF2RA, CSF1R, CXCR4, BCL6, CXCL2, IL6, and CD11b genes expression were tested by RT-PCR. During the process of amplification, genes were added to 2X qPCR / RTD-PCR Master mix E4 (SYBR Green AB kit), forward primer and revers primer (Metabion, Germany), cDNA and ddH_2_O. Reactions were performed in Real-Time PCR device (AB Applied Biosystems, stepone Real-time PCR).GAPDH gene was used as an internal control. Also two main cytokines, SDF-1 and GM-CSF expression level was measured in MSCs by qualitative PCR in normal condition.The pairs of primers that used for gene amplification are presented in Table 1.

### Statistical Analysis

The results data were reported as mean ± S.D and were analyzed using GraphPad Prism v 5.00 (GraphPad Software, Inc., La Jolla, CA). Student’s t-test were used for the presented results. P value < 0.01 was considered statistically significant.

**Table 1:** Primers for Real-Time Polymerase Chain Reaction

**Gene**	**Forward primer**	**Revers primer**
CD14	CTGGAACAGGTGCCTAAAGGAC	GTCCAGTGTCAGGTTATCCACC
CXCL8	GAGAGTGATTGAGAGTGGACCAC	CACAACCCTCTGCACCCAGTTT
TNFRSF11A	GCTCAACAAGGACACAGTGTGC	CGCATCGGATTTCTCTGTCCCA
CSF2RA	CCTGTCAGGATTAACGTCTCGC	CATTGCTGGGAGGGTTGAATCG
CSF1R	CCTGTCAGGATTAACGTCTCGC	CATTGCTGGGAGGGTTGAATCG
CXCR4	CTCCTCTTTGTCATCACGCTTCC	GGATGAGGACACTGCTGTAGAG
BCL6	CATGCAGAGATGTGCCTCCACA	TCAGAGAAGCGGCAGTCACACT
CXCL2	GGCAGAAAGCTTGTCTCAACCC	CTCCTTCAGGAACAGCCACCAA
IL6	AGACAGCCACTCACCTCTTCAG	TTCTGCCAGTGCCTCTTTGCTG
CD11b	GGAACGCCATTGTCTGCTTTCG	ATGCTGAGGTCATCCTGGCAGA
GAPDH	ACCCATCACCATCTTCCAGGAG	GAAGGGGCGGAGATGATGAC
GM-CSF	GGAGCATGTGAATGCCATCCAG	CTGGAGGTCAAACATTTCTGAGAT
SDF-1	CTCCTCTTTGTCATCACGCTTCC	GGATGAGGACACTGCTGTAGAG

## Results

### Microscopic examination of U937 cells morphology

**Figure 1:**
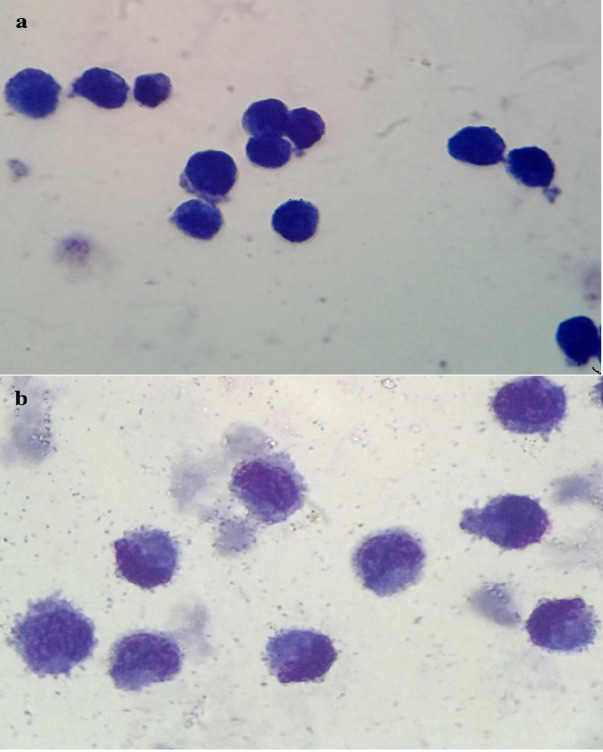
Morphology of U937 cells. a. control b. U937 cells treated with VitD3(5×10^-8^M) (magnitude ×100).

Wright Giemsa staining was performed on U937 cells as control and group II, U937 cells treated with VitD3, after 7 days of incubation. The group II cells acquired a monocytic morphology; kidney-shaped nucleus and blue*-*grey cytoplasm. Cytoplasmic vacuoles were seen in some cells (Figure 1).

### CD14 expression increased in U937 cells co-culture with MSCs and conditioned medium

**Figure 2:**
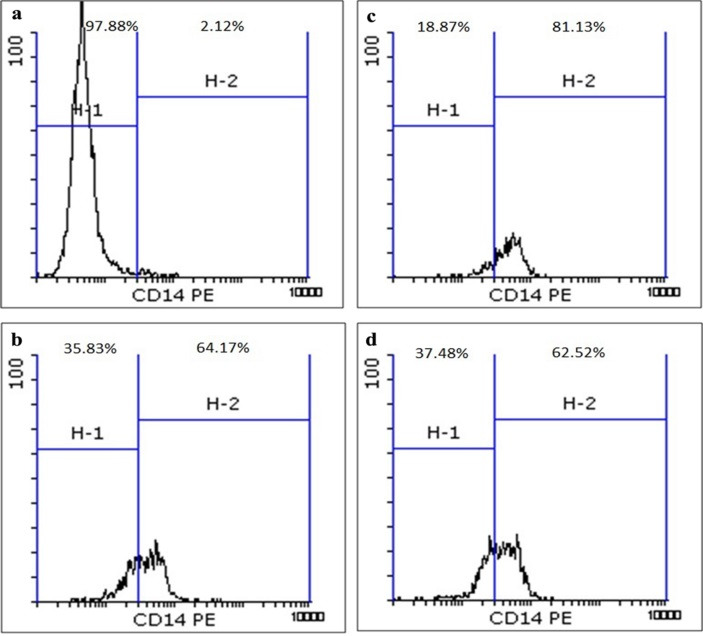
Flow cytometric analysis of monocytic marker differentiation after 24 hours. a. Group I, b. Group II, c. Group III, d. Group IV.

Flow cytometric evaluation confirmed an increase in the percentage of CD14 marker expression, one of the main monocytic differentiation markers, in the groups II, III and IV cells and the induced U937 cells differentiation. In the group III, U937 cells co-cultured with MSCs, there was a higher increase in CD14 marker expression compared to other groups (Figure 2).

### Effect of U937 cells co-culture with MSCs and conditioned medium on CD14, CXCL8, TNFRSF11A, CSF2RA, CSF1R, CXCR4, BCL6, CXCL2 Genes expression

**Figure 3:**
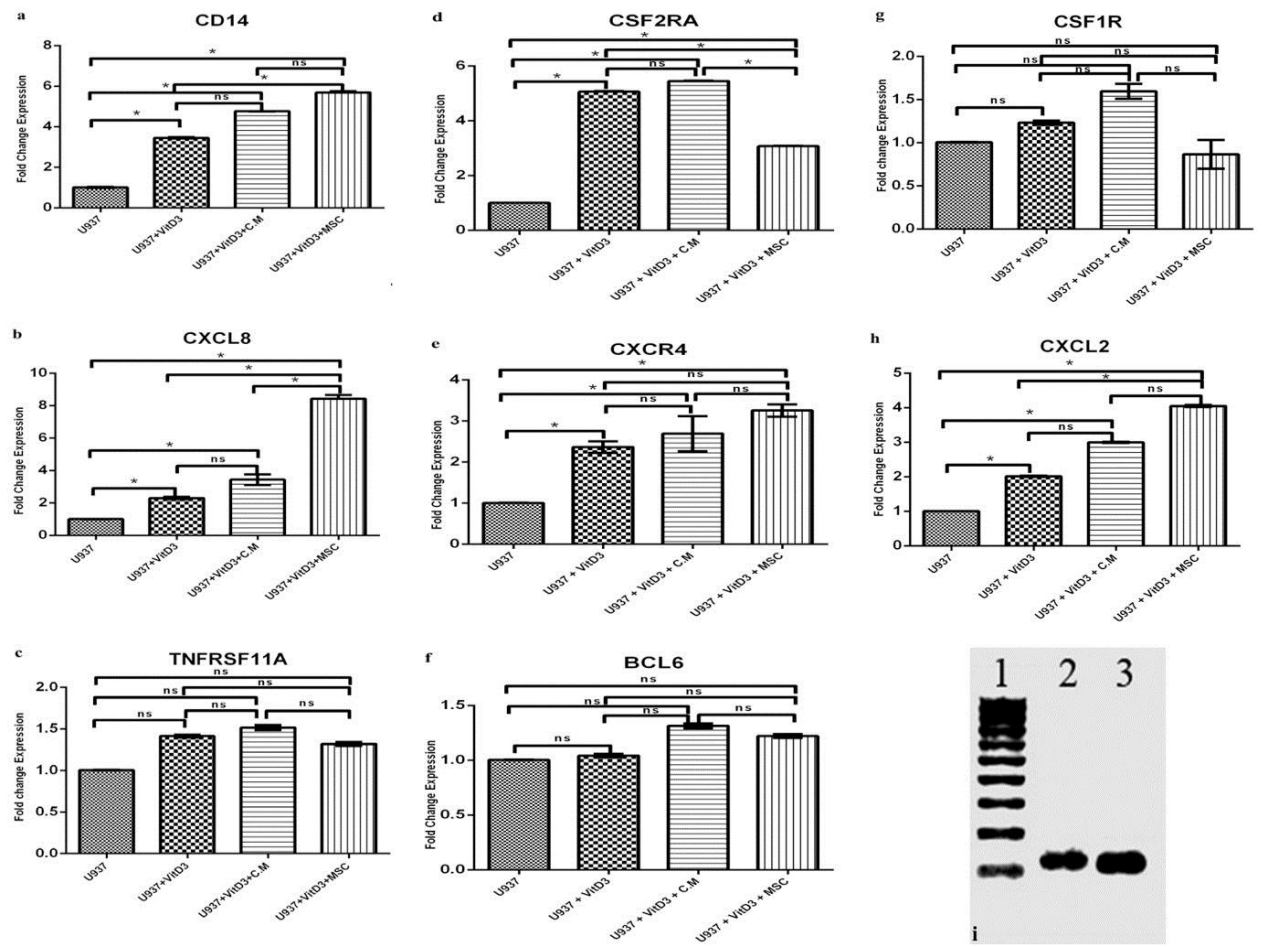
Genes expression during differentiation of U937 cells after 24 hours‏ . a. CD14 gene expression, b. CXCL8 gene expression, c. TNFRSF11A gene expression, d. CSF2RA gene expression, e. CXCR4 gene expression, f. BCL6 gene expression, g. CSF1R gene expression, h. CXCL2 gene expression. i. SDF-1 and GM-CSF genes expression in MSCs co-cultured with U937 cells after 24 hr ( lane1: DNA ladder 100bp, lane2: GM-CSF, Lane3: SDF1). *P<0.01, ns: non significant.

Real-time PCR analysis showed a marked increase in the CSF2RA gene, and enhanced CXCL8, CXCR4, CXCL2 genes expression in the group II cells (P<0.01). In the group III, U937 cells co-cultured with MSCs, there was a significant increase in CXCL8, and CXCL2 genes expression and enhanced CSF2RA, and CXCR4 genes expression (P<0.01). In the group IV cells, there was a marked increase in CSF2RA, CXCL8 genes expression and enhanced CXCR4, CXCL2 genes expression (P<0.01). In all related groups, CD14 gene was considerably increased and there was no significant increase in TNFRSF11A, BCL6, and CSF1R genes expression. IL6 and CD11b genes were also examined and there was no expression in all groups (Figure 3).

## Discussion

MSCs are an important cellular component of the hematopoietic microenvironment. They can support hematopoiesis by providing numerous cytokines and growth factors and via expression of cell adhesion molecules that are important for cell-to-cell interaction.^6^ Previous studies show that a feeder layer of MSCs, plays a vital role in the development and regulation of the differentiation of hematopoietic stem cells.^15,16^ Some studies have shown that MSCs as a feeder layer maintain HSCs in an undifferentiated state.^17^ Another study showed that MSCs modulate U937 cells proliferation and have a main role in determining the sensitivity of leukemic cells to treatment.^18^ It has been shown that close contact between mesenchymal stem cells and hematopoietic stem cells support the maturation of hematopoietic stem cells into red blood cells.^19^ Angelopoulou et al. showed that co-tranplantation of MSCs enhance differentiation of CD34^+^-selected HSCs toward the myeloid series.^20^ The result of this research showed that MSCs enhance the differential effect of VitD3 on U937 cells and also U937 cells co-cultured with MSCs are superior to U937 cells cultured with the condition medium, in supporting the monocytic U937 cells differentiation. This indicates that cell-cell contact between U937 and MSCs are more effective than factors secreted by MSCs and increased cell adherence can also promote the effect of MSC. Cytokines and growth factors including SCF, CXCL12(SDF1), GM-CSF, and M-CSF, are known to be secreted by MSC.^8^ Loss of SCF secretion by supporting cells and MSCs or disruption of the SCF receptor on HSC leads to defect in hematopoiesis, indicating the essential role of MSC in the HSC niche.^21^ CXCL12(SDF-1) chemokine regulates the release of HSCs and their migration from bone marrow to the vascular niche and bone marrow homing of HSCs.^22-27^ SCF and CXCL12 secreted by MSC binding to c-Kit and CXCR4 respectively on the HSC, lead to the activation of different signaling pathways including the Phosphatidylinositol3-kinase (PI3K).^28-31^ Activation of the PI3K-Akt-1 signaling pathway plays an important role in VitD3-stimulated cells differentiation. Studies show that the PI3K-Akt-1 signaling pathway is important to stimulate monocytic differentiation by VitD3 and protect against apoptosis.^32^ Our data showed that increased amount of CXCR4 receptor could be find in U937 cells which co-cultured with MSCs, also we found that MSCs express SDF-1 gene highly. These findings together suggest one of putative pathways of MSCs effect on supporting monocytic differentiation of U937. Already, it has been shown that CXCL12 chemokine signaling pathway supports *ex vivo* proliferation and expansion of HSC.^27^ The results of this study show that expression of the CXCR4 gene, and CXCL12 receptor increases because the MSCs secrete CXCL12, maybe this is one of the U937 cell differentiation factors. GM-CSF and VitD3 induce differentiation of U937 cells synergistically, through up-regulation of c-fos mRNA and down-regulation of c-myc mRNA and shift in cell cycle.^33^ MSC attachment to HSC via N-cadherin and β-integrins leads to increased expression of Notch receptors on the MSC and upregulated Notch signaling pathway in HSC. Moreover, soluble cytokines from MSCs such as SCF, SDF1 bind to their receptors on the HSC surface and support the growth and differentiation of HSC.^7^ The Notch signaling pathway plays a role in the survival and proliferation of HSCs.^34^ This study shows that MSCs can regulate the differentiation of U937 cells and MSCs exert their effects more efficiently through adhesion molecules, such as N-cadherin and *β*-integrins, in cell-cell contact. Reduction in HSCs can be observed after the depletion of MSCs in BM, at least in part to an increase in HSCs mobilization towards extra medullary sites.^35^ A study showed that MSCs in co-culture with monocyte cells‏ isolated from peripheral blood mononuclear cells, suppressed the initial differentiation of monocytes into dendritic cells, but this effect was reversible.^36^ Hao-Ping Sun et al. showed that a feeder layer of human umbilical cord blood-derived stromal cells, can better support than the human umbilical cord blood-derived mesenchymal stem cells in hematopoiesis stem cells differentiation into the myeloid lineage cells *in vitro.*^37^ The current study demonstrated that MSCs support the differential effect of Vit D3 on U937 cells. The cellular and molecular mechanisms of the positive effect of MSCs on HSCs differentiation remain unclear. The effect of mesenchymal stem cells on the differentiation of U937 cells requires more studies in future.

## Conclusion

One potential application for MSCs is their use in HSC transplantation that is far ahead of other indications. Currently injecting MSCs are used as a routine method in bone marrow transplantation; therefore, evaluation of MSCs and their effects on HSC is essential. The result obtained in this study partly confirms that MSCs could increase monocytic differentiation in the U937 cell line model. Although, it was found that some monopoesis related genes were modulated in the MSC co-culture. However, the definite mechanism of the phenomenon should be evaluated using more extended experiments and in primary hematopoetic cells differentiation model co-cultured with MSCs.

## Acknowledgments

We highly appreciate East Azerbaijan Province Blood Transfusion Headquarter to provide laboratory facility of this research also we would like to thank Tabriz University of Medical Sciences for financial supporting this research.

## Ethical Issues

Not applicable.

## Conflict of Interest

Authors declare no conflict of interest in this study.
